# Fine-Scale Dietary Polyphenol Intake Is Associated with Systemic and Gastrointestinal Inflammation in Healthy Adults

**DOI:** 10.1016/j.tjnut.2024.08.010

**Published:** 2024-08-18

**Authors:** Stephanie MG Wilson, Andrew Oliver, Jules A Larke, José J Naveja, Zeynep Alkan, Joseph M Awika, Charles B Stephensen, Danielle G Lemay

**Affiliations:** 1 United States Department of Food and Agriculture, Agricultural Research Service Western Human Nutrition Research Center, Davis, CA, United States; 2 Texas A&M AgriLife Research, Institute for Advancing Health Through Agriculture, College Station, TX, United States; 3 3rd Medical Department, University Medical Center, Johannes Gutenberg University Mainz, Mainz, Germany; 4 Institute of Molecular Biology gGmbH, Mainz, Germany; 5 Department of Nutrition, University of California, Davis, Davis, CA, United States

**Keywords:** food composition, dietary assessment, gastrointestinal health, systemic inflammation, bioinformatics, polyphenol consumption, immunity, machine learning, computational biology, food database

## Abstract

**Background:**

Polyphenols are dietary bioactive compounds, many of which have anti-inflammatory properties. However, information on the intake of dietary polyphenols at the class and compound levels and their associations with gastrointestinal (GI) and systemic inflammation is lacking.

**Objectives:**

Estimate dietary polyphenol intake in healthy adults and examine its relationship with GI and systemic inflammation markers.

**Methods:**

Healthy adults (*n* = 350) completed the United States Department of Agriculture Nutritional Phenotyping Study, an observational, cross-sectional study balanced for age, sex, and body mass index. Dietary intake, assessed via multiple 24-h recalls, was ingredientized and mapped to FooDB, a comprehensive food composition database. Dietary polyphenol intake (total, class, compound) was estimated and examined for its relationship to GI and systemic inflammation markers using linear models and random forest regressions.

**Results:**

Mean total polyphenol intake was ∼914 mg/1000 kcal/d with flavonoids as the greatest class contributor (495 mg/1000 kcal/d). Tea, coffee, and fruits were among the largest food contributors to polyphenol intake. Total polyphenol intake was negatively associated with the GI inflammation marker, fecal calprotectin (β = −0.004, *P* = 0.04). At the class level, polyphenols categorized as prenol lipids (β = −0.94, *P* < 0.01) and phenylpropanoic acids (β = −0.92, *P* < 0.01) were negatively associated with plasma lipopolysaccharide-binding protein, a proxy for GI permeability. Food sources of these two classes included mainly olive products. We further detected a positive association between C-reactive protein and polyphenols in the “cinnamic acids and derivatives” class using hierarchical feature engineering and random forest modeling.

**Conclusions:**

Even in healthy adults, dietary polyphenol intake was negatively associated with GI inflammation and intake of prenol lipids and phenylpropanoic acids was negatively associated with GI permeability. Relationships between polyphenol intake and inflammatory outcomes varied with the resolution—total, class, compound—of polyphenol intake, suggesting a nuanced impact of polyphenols on GI and systemic inflammation.

This trial was registered at clinicaltrials.gov as NCT02367287.

## Introduction

The consumption of foods rich in phenolic compounds (henceforth referred to as polyphenols) may help reduce systemic inflammation and other cardiovascular disease risk factors, a benefit largely attributed to the additive and synergistic capacity of polyphenols to act as antioxidants and anti-inflammatory agents [[1], [2], [3]]. Dietary intake of polyphenols in adults is dependent on personal and sociocultural food preferences with the average estimated total polyphenolic intakes ranging from ∼400 mg/d [4] to >1000 mg/d [5,6].

Once ingested, the structure and physiochemical properties of the polyphenol influence its absorption, metabolism, and elimination kinetics [4,[7], [8], [9]]. Total polyphenol absorption in the small intestine is estimated to be 5%–10% with the remaining 90%–95% of polyphenols passing to the large intestine for microbial enzymatic action. Polyphenols more likely to reach the large intestine include those with a higher molecular weight, glycosides, polymeric forms, polyphenols bound to plant cell wall polysaccharides [10], and phase I and phase II polyphenol metabolites effluxed into the small intestine from enterocytes. Polyphenols and their derivatives can also exert biological effects in the large intestine, including antimicrobial activity [11,12], suppression of inflammatory signaling [13,14], and enrichment of microbial producers of short chain fatty acids [15] shown in humans, and elevated mucus and IgA production shown in murine models [16]. These findings collectively suggest that polyphenols may contribute to an improved gastrointestinal (GI) environment in a manner potentially sensitive to differences in polyphenol absorption, distribution, metabolism, and excretion.

There is an increasing need to understand how poorly characterized phytochemicals in food, termed nutritional “dark matter” [17], influence health outcomes. Publicly available food composition databases are frequently utilized to quantify nutrients but are limited in their coverage of phytonutrients such as polyphenols. Given the food and compound coverage limitations of a singular database, a combination of multiple databases has been suggested to establish the polyphenol composition of foods [18]. FooDB is a freely available online food composition database that aims to remedy the lack of a central system for food phytochemical composition data [19] by curating multiple food composition databases, such as Phenol-Explorer [20] and USDA special interest databases [21,22], and experimental data on specific foods from numerous publications. As over 3000 polyphenols have been identified within FooDB [23], the use of FooDB has untapped potential in the effort to provide more accurate estimates of dietary polyphenol intake, and consequently better understand their role in human health.

The anti-inflammatory impact of polyphenols in adults has been largely studied in the context of systemic inflammation [2]. Given most ingested polyphenols reach the large intestine, it is imperative to also examine the potential benefits of polyphenols on markers of GI inflammation and assess if there are potential differential impacts between polyphenols. In this secondary analysis of the USDA Western Human Nutrition Research Center Phenotyping Study, we assessed the relationship between dietary polyphenol intake and markers of GI inflammation, GI permeability, and systemic inflammation. We hypothesized that GI inflammation, GI permeability, and systemic inflammation were inversely correlated with polyphenol intake. To accomplish this, we first estimated the polyphenol content in 24-h diet recall data by mapping recent dietary intake to the FooDB database. Next, we summarized polyphenol intake at various resolution levels: total intake, taxonomic class intake, and intakes across taxonomic levels as polyphenol bioavailability and bioefficacy can differ on the basis of their physiochemical properties. Lastly, we assessed the strength and directionality between polyphenol intake and GI inflammation, GI permeability, and systemic inflammation.

## Methods

### Study design

This study is a secondary analysis of the USDA Nutritional Phenotyping Study, an observational, cross-sectional study conducted by the USDA Western Human Nutrition Research Center from May 2015 to July 2019 [24]. The study protocol was approved by the Institutional Review Board at the University of California, Davis and prospectively registered at ClinicalTrials.gov (NCT02367287).

### Participants

Generally healthy adults were recruited from around the Davis, California area. Eligible adults were 18–65 y old, male or female, and had a BMI between 18.5 and 45 kg/m^2^. Participants were recruited using a sampling scheme with 18 categories determined by age, sex, and BMI to balance enrollment across seasons and years. Adults were excluded from participation if they met one of the following criteria: pregnancy, lactating, had a known egg-white protein allergy, systolic blood pressure >140 mm Hg or diastolic blood pressure >90 mm Hg measured on 3 occasions, had a diagnosed active chronic disease (including GI disorders) treated with medication, had a recent (<4 wk) minor or major (<16 wk) surgery, had recent antibiotics (<4 wk), had a recent hospitalization (<4 wk), or used prescription medications affecting outcomes of interest. The study design article includes further details about primary analyses, exclusion criteria, and the sampling scheme [24]. In total, 393 participants enrolled into the study with 350 adults completing the study with ≥2 dietary records that passed quality control. The STROBE flow diagram is available in Supplemental Figure 1.

### Outcomes of interest

The primary outcomes of interest include systemic inflammation, GI inflammation, and GI permeability. Fecal calprotectin (CAL), fecal myeloperoxidase (MPO), and fecal neopterin (NPO) were selected as markers of GI inflammation [25]. Plasma LPS binding protein (LBP) was selected as a marker of GI permeability [26], and plasma C-reactive protein (CRP) was used to assess systemic inflammation [27].

### Lifestyle

Demographic questions were administered as an electronic survey through REDCap [28] with access provided by the UC Davis Clinical and Translational Science Center. Demographic information relevant to this study included education level received, household income, and race.

### Diet assessment

Dietary intake data (24-h recalls or food records) were collected using the Automated Self-Administered 24-h (ASA24®) Dietary Assessment Tool, versions 2014 and 2016, developed by the National Cancer Institute. The ASA24 utilizes the Food and Nutrient Database for Dietary Studies (FNDDS).

Participants went through a training dietary recall with a study staff member during which they were presented with measuring cups and spoons to demonstrate serving sizes. They were then asked to self-complete three 24-h dietary recalls at home within the 10–14 d between their initial intake and blood collection visit as described previously [24]. Dietary recall data were cleaned with quality control methods developed for ASA24 recalls [29]. ASA24 total healthy eating index (HEI)-2015 scores were calculated to assess dietary quality. Participants with ≥2 at-home dietary recalls were included for dietary analyses (*n* = 350).

### “Ingredientization” of dietary data

Foods with multiple ingredients in ASA24 data were disaggregated into underlying individual ingredients using a semiautomated process termed here as “ingredientization” and outlined previously [30]. The “ingredientization” process began by first mapping ASA24 food and beverage items to 2017–2018 FNDDS Ingredients data and calculating caloric and nutrient intakes. Four, 5, and 6-digit food codes are based on Food Data Central codes whereas 8-digit codes are specific food codes to FNDDS. Eight-digit food codes were broken down in an iterative process to find the underlying ingredients beneath the broader food code using built-in FNDDS recipes. As an example, “Spaghetti sauce with added vegetables” with the FNDDS code 74404020 would be broken down into the ingredients: marinara, mushrooms, and peppers. Food codes that could not be broken down further constituted the final ingredient list. Final FNDDS ingredient mappings are provided in the supplemental files of the reference article [30]. Vitamin D was removed from the final ingredient list as the only ingredient-nutrient code provided by FNDDS. The amount of ingredient consumed was calculated as food amount multiplied by ingredient proportion (ingredient weight divided by total food weight).

### FooDB polyphenol identification

Though polyphenols in FooDB were previously identified in 2018 [23], we rescreened FooDB for polyphenols to account for database updates. Food composition data on over 70,000 food-derived compounds were gathered from the FooDB database (version 1) downloaded September 2022 [31]. Polyphenol identification was performed by utilizing structural data as outlined previously [23]. Briefly, simplified molecular input line entry system (SMILES) data from the FooDB compound file were read into the cheminformatics software RDKit [32]. Salts were removed and rings aromatized before SMILES were converted to the canonical SMILES form according to the default settings in RDKit KNIME (Version 4.1.3) nodes. The following SMILES arbitrary target specification (SMARTS) substructure pattern was used to identify polyphenolic compounds: [OX2H][cr6].[OX2H][cr6]. The SMARTS pattern used defines a polyphenol as a compound with an aromatic ring of 6 members bound to ≥2 nonsubstituted hydroxyl groups. Consequently, mono-hydroxylated phenolic compounds were not included in this definition. Of the 70,477 compounds in FooDB, 3080 compounds were identified as polyphenolic.

Within downloaded files, discrepancies between compound name and structure were identified in compounds indexed to a single experimental source examining bovine milk. Thus, all milk compounds and their concentrations were harvested from FooDB.ca, which is more regularly updated, using taxonomic xpaths with the R packages *rvest* version 1.0.3 [33] and *xml2* version 1.3.4 [34]. The harvest yielded 2216 compounds in milk that were rescreened for a polyphenolic structure. Sixteen polyphenols were identified which replaced the original 42 compounds in milk detected from downloadable files. The polyphenol compound count after the milk corrections in FooDB was 3063.

### Polyphenol taxonomy

Taxonomic data on polyphenols were collected from downloadable FooDB files. Polyphenols without complete taxonomic classification data were identified and their taxonomic data harvested from FooDB using taxonomic xpaths with rvest version 1.0.3 [33] and xml2 version 1.3.4 [34]. Taxonomic classification of compounds in FooDB are derived from ClassyFire, an automated chemical taxonomic classification application [35].

### Ingredient mapping

All original food source descriptions in FooDB (orig_food_ common_name) and ingredient descriptions from the “ingredientized” dietary recalls underwent a typical text cleaning process, which involves the removal of upper case, punctuation, and stop words. As FooDB entries are referenced, original food source codes embedded within FooDB were first matched to ASA24 ingredient level dietary data. Matches were confirmed by string similarity. Food codes that did not have an original source match went through natural language processing, which leveraged dependency parsing of ingredient descriptions and macronutrient data to improve automated searching of FooDB ingredients. Dependencies were generated using the medium English pipeline by spaCy (Version 3.3.1) in Python. A dependency-based search was performed to identify possible matches, with match quality assessed using text similarity. Poor matches were rescreened using text similarity ranking and macronutrient ranking, with the lowest rank indicating the best match. A graphical overview of database handling and mapping is provided in Supplemental Figure 2.

### Estimation of polyphenol intake

Polyphenol concentrations of original foods were standardized to mg per 100 g and aggregated under the FooDB-assigned food level ID to match concentrations shown on FooDB.ca. As an example, “cauliflower,” “cauliflower, raw,” and “cauliflower, cooked, boiled, drained without salt” are original food names that were aggregated under “Cauliflower” (FOOD00031). Before aggregation, content entries from Dr. Duke’s Phytochemical and Ethnobotanical Database [36] were manually reviewed to include commonly consumed food parts. Additionally, the Duke database contained entries for coffee and tea reported as dry weight which were adjusted according to standard brewing ratios. The Duke-specific adjustments affected 17 of the 333 quantified compounds within “Tea” (FOOD00038) and 0 of 26 quantified compounds within “Arabica Coffee” (FOOD00059). Foods with no predicted polyphenols in FooDB, including most meats and seafood, were assigned a zero concentration.

Application of retention factors is recommended in polyphenol estimation for improved accuracy as factors capture the increase or decrease in abundance of nutrients from food cooking and processing. Although FooDB does not contain retention factors, it does preserve underlying source IDs. Using the ID cross linkage, we mapped foods between FooDB and Phenol-Explorer [20] to apply retention factors to FooDB foods. Content entries derived from Phenol-Explorer were extracted from FooDB and matched to their corresponding Phenol-Explorer food ID within the retention factor data file (Supplemental Figure 2). Retention factors were multiplied against original food content. Polyphenol intake by compound and food was calculated as the average across all available recalls. With a high number of quantified foods across participants (mean ± SD, 94% ± 2.4%), no additional filtering was performed. Chinese tannin and alpha-viniferin were identified as outlying compounds on the basis of mean intakes and were removed before analyses.

### Blood collection and processing

Participants were asked to refrain from nutritional and herbal supplements for a 3-d washout leading up to fasting blood collection. Fasting blood samples are representative of a steady state as polyphenols are regularly consumed and their microbial metabolites have kinetic profiles that may last up to 48-72 hours systemically [37]. Participants were provided a standard evening meal (30% kcal from fat, 55% kcal from carbohydrate, 15% kcal from protein) consisting of egg-stir fry, potato soup, and commercially available raspberry sorbet and lemonade to consume between 18:00 and 19:00. A fasting blood sample was collected from participants after an overnight 12-h fast [24]. Blood was collected by venipuncture performed by a certified phlebotomist. After collection, tubes were placed on ice, then centrifuged at 1300 × *g* at 4°C for 10 min. Aliquots were prepared immediately and stored in Cryo-Store® vials at −80°C until analysis.

### Systemic inflammation and intestinal permeability

CRP is a commonly assessed marker of systemic inflammation and is predictive of chronic disease risk [27]. Thus, high-sensitivity CRP was determined from EDTA plasma with an electrochemiluminescent multiplex assay (V-PLEX Vascular Injury Panel 1) using the MESO Scale Discovery sector imager. Plasma LBP, used in this healthy cohort as a proxy for intestinal permeability [26], was determined using the LBP (Human) ELISA (Abnova; #KA0448) as has been previously described [38]. Samples that met quality control were included for CRP (*n* = 348) and LBP (*n* = 350) analysis.

### Stool collection

Participants were asked to collect a single whole stool specimen as close to their scheduled blood collection visit as possible. Participants were provided at-home stool collection kits and instructions. Samples were enclosed in a Ziploc bag within a hard plastic lidded container and returned in a chilled cooler for same-day processing. Stool collection and processing times were recorded. Stool samples with >24 h between collection and processing were excluded from analysis (*n* = 30). A Stomacher paddle blender was used to homogenize samples before flash-freezing on dry ice and storage at −80°C.

### Gastrointestinal inflammation

CAL, MPO, and fecal NEO are indicators of GI inflammation [25]. Concentrations of CAL, MPO, and NEO in homogenized stool samples (*n* = 307) were determined using the input sample weights and the corresponding dilution factor. Determination of CAL and MPO [38] and NEO [30] with ELISAs have been described previously. The following kits were used: CAL ELISA (Immundiagnostik; #K6927), MPO ELISA (Immundiagnostik; #KR6630), and NEO ELISA (B·R·A·H·M·S/ThermoFisher; #14-HD-99.1).

### Statistical analysis

All analyses and visualizations were generated using RStudio (2023.6.1.524) running R version 4.2.2 unless specified otherwise.

We examined the relationship between energy-adjusted total polyphenol intake (mg/1000 kcal) and our main covariates (age, sex, and BMI) using a rank-based estimation regression. Regressions were performed using Rfit version 0.24.2 [39]. As plant-rich dietary patterns are already considered healthy, it is difficult to separate the benefits of polyphenols from other co-occurring beneficial compounds. Thus, we also modeled outcomes with and without total dietary fiber and ASA24 total HEI score. Age, BMI, sex, fiber, and HEI were treated as continuous variables. Top food contributors to total polyphenol intake were foods in the highest quartiles of both polyphenol intake and frequency for the cohort.

We assessed the relationship between energy-adjusted total daily polyphenol intake (mg/1000 kcal) and covariates, with and without fiber and total HEI score on markers of inflammation (CRP, LBP, CAL, MPO, NEO). Relationships were analyzed using rank-based estimation regression, which performs a nonparametric estimate of regression coefficients.

To assess the relationship of each polyphenol class intake on each marker of inflammation, we performed linear models with and without fiber and total HEI score. *P* values were adjusted using the false discovery rate multiple testing correction. Polyphenol class intakes were normalized according to recommendations from bestNormalize in the bestNormalize R package (version 1.9.0). Forty-two classes had >80% of participants with zero intake and were removed from analyses.

### Machine learning analyses

Machine learning can find useful patterns in data and reveal relationships between dietary features and health outcomes of interest. Thus, machine learning regressions were used to investigate the influence of individual polyphenols on markers of inflammation as machine learning can also handle many predictors and nonlinear relationships. In this nonhypothesis testing paradigm, which is used after specific hypotheses have been tested, we specifically examined participants below subclinical thresholds of inflammation, which was previously shown to better predict relationships between dietary patterns and inflammation in healthy adults [40]. Subclinical inflammation was defined as >10 mg/L for CRP and >100 μg/g for CAL. The acute inflammation threshold for CAL is 50 μg/g with fresh stool; however, a higher threshold was used per kit instructions as elevated CAL was expected because of cell lysis during sample freezing and thawing. MPO, NEO, and LBP were not analyzed as there is not an established threshold delineating subclinical inflammation.

Reducing the number of features can improve the performance of machine learning models. Thus, polyphenol taxonomy was leveraged to reduce the number of polyphenol features by using TaxaHFE, a hierarchical feature engineering software [41]. TaxaHFE utilizes the pairwise correlation structure between a taxon and its descendants to trim descendants above a correlation threshold, set in this study to 0.95. Remaining taxa pass through a random forest model against a continuous or categorical outcome, where taxa are further trimmed on the basis of average feature importance. In this study, polyphenol features were engineered to maximize their predicative power of continuous inflammation outcomes.

Reduced polyphenol feature sets were combined with other covariates listed in Supplemental Table 1 as inputs into a random forest regression model facilitated by the dietML program (github.com/aoliver44/nutrition_tools), which incorporates “best practices” in machine learning analyses. In brief, an initial 80–20 train–test split was performed followed by data preprocessing evaluation inside 10-fold repeated cross-validation. Shapley Additive exPlanations values were calculated and used to explain feature importance.

## Results

### Participant characteristics

Overall, the average age was 40 ± 14 y (mean ± SD) and average BMI 27 ± 5 kg/m^2^. Sex had balanced representation with 186 females and 164 males. Over 60% of subjects identified as white, 14% identified as Hispanic or Latinx, and 11.7% identified as Asian or members of an Asian subgroup. Most subjects reported having at least a bachelor’s degree whereas there was a diversity of reported household incomes. Table 1 [61,62] provides counts for age, sex, and BMI categories, as well as counts for race, level of education received, and annual household income.

**TABLE 1:** Characteristics of the USDA Phenotyping Cohort (*n* = 350).

Characteristic	*n*
Age (y)	
18–33.99	121
34–49.00	117
50–65	112
BMI (kg/m^2^)	
18.5–24.99	135
25–29.99	125
30–39.99	90
Sex	
Female	186
Male	164
Race	
American Indian or Alaska Native	1
Asian	33
Black	15
East Asian	2
Hispanic or Latinx	49
Middle Eastern	5
Native Hawaiian or Pacific Islander	1
Race declined	3
South Asian	2
Southeast Asian	4
Two or more races	21
White	214
Education level	
Primary school (first–sixth)	1
High school, GED, or equivalent	14
Some college/Associate’s degree	89
Bachelor’s degree	124
Graduate degree, nondoctoral	80
Doctorate degree	42
Household Income	
$0–$19,999	43
$20–49,999	48
$40–59,999	46
$60–79,999	44
$80–99,999	34
>$100k	101
Decline to respond	31
NA	3

Abbreviations: BMI, body mass index; NA, not applicable.

Race, education level, and household income were self-reported by participants. Additional clinical information, including anthropometrics and blood lipid profiles, can be found in other analyses of this cohort [61,62].

### Total polyphenol intake

Mean total polyphenol intake in our cohort of 350 healthy adults was ∼914 ± 50 (SE) mg per 1000 kcal/d. Average total polyphenol intake increased with age (β = 7.6, *P* < 0.0001), decreased with BMI (β = −13.6, *P* = 0.01), and was lower in males than females (β = −138.5, *P* = 0.01), with the relationship and directionality for age (β = 5.4, *P* = 0.009) and sex (β = −142.9, *P* = 0.01) persisting after adjustment for average total fiber intake (β = 2.1, *P* = 0.45) and total HEI score (β = 10.2, *P* < 0.001). BMI (β = −3.1, *P* = 0.60) was not found to relate to total polyphenol intake after adjustment for fiber intake and total HEI score.

Table 2 details mean intakes of total polyphenols and polyphenols classes from 24-h dietary recalls across our cohort and by age, sex, and BMI bins. Of the 3063 polyphenols identified within FooDB, we detected 1483 polyphenols in our dietary data which represent 57 classes. Of the 57 classes, 27 had concentrations in FooDB that were quantified. Participants consumed an average of 44 ± 4 (SD) polyphenolic classes. Polyphenol intakes at the class level followed similar patterns across covariate bins, with flavonoids consistently being the largest contributor to total polyphenol intake with an average flavonoid intake of ∼495 ± 38 (SE) mg per 1000 kcal/d across our cohort.

**TABLE 2:** Polyphenol class contributions to average total energy-adjusted polyphenol intake.

		Age (y)			Body mass index (kg/m^2^)			Sex	
Polyphenol class (mg/1000 kcal)	All (*n* = 350)	18–33.99 (*n* = 121)	34–49.00 (*n* = 117)	50–65 (*n* = 112)	18.5–24.99 (*n* = 135)	25–29.99 (*n* = 125)	30–39.99 (*n* = 90)	Female (*n* = 186)	Male (*n* = 164)
Flavonoids	495	480	423	586	478	562	427	564	417
Organo-oxygen compounds	125	99	132	146	107	142	130	145	103
Benzene and substituted derivatives	98	65	116	115	106	148	15	84	114
2-arylbenzofuran flavonoids	80	50	89	104	93	97	39	87	72
Cinnamic acids and derivatives	35	28	37	42	32	38	37	41	29
Alcohols and polyols	30	20	25	47	28	35	28	37	23
Tannins	23	20	21	28	23	26	18	26	19
Isoflavonoids	18	18	18	18	19	18	16	18	19
Phenols	8	7	8	9	9	8	6	8	8
Other	1	0	0	0	0	0	0	0	0

Dietary intakes of healthy adults were estimated through 24-h dietary recalls and are presented here by class across age, BMI, and sex bins used in the study sampling scheme. “Other” included the remaining polyphenol classes where intake was >0, which includes: aporphines, aryltetralin lignans, benzopyrans, carboxylic acids and derivatives, coumarins and derivatives, cyclobutane lignans, depsides and depsidones, diarylheptanoids, dibenzylbutane lignans, fatty acyls, furanoid lignans, indanes, lignan lactones, linear 1,3-diarylpropanoids, naphthalenes, phenylpropanoic acids, prenol lipids, and stilbenes.

### Food contributors to total polyphenol intake

The top food sources of polyphenols, average food amount, and the average polyphenol amount from each are presented in Table 3. Tea was a major food source of polyphenols and consumed by ∼41% of our cohort. Other beverages including coffee and wine also contributed to polyphenol intake. Fruits such as grapes, oranges, apples, blueberries (*Vaccinium caesariense*), and strawberries were additional rich sources of polyphenols. “Sweet orange” oranges notably included orange juice consumption. Wheat was detected as a polyphenol source by “ingredientizing” foods with multiple ingredients, stemming from foods including breads, breading, desserts, and tortillas. The main compound associated with wheat was pelargonidin 3-(6''-succinyl-glucoside). The common bean also was a contributor to daily total polyphenol intake.

**TABLE 3:** Top 10 food contributors to estimated total polyphenol intake.

Rank	Food	User frequency	Average polyphenol intake (mg)	Average food amount consumed (g)
1	Tea^1^	143	1290	401
2	Common grape	121	442	30
3	Sweet orange	105	327	151
4	Coffee^1^	235	253	322
5	Apple	146	136	140
6	Wheat	184	116	24
7	Grape wine	88	114	245
8	*Vaccinium*^2^	106	40.5	66
9	Strawberry	117	38	76
10	Common bean	203	24.9	69

Top contributors to total polyphenol intake in both the highest quartiles of user frequency and polyphenol intake, with rank determined by average polyphenol intake per day. Polyphenol intake and food amount consumed represent the average across all participants.

1 As brewed.

2 Genus includes blueberries, cranberries, and huckleberries.

### Relations of total polyphenol intake with inflammation

Total polyphenol intake was negatively related to CAL (*P* = 0.04) after accounting for age, BMI, and sex. When total fiber and total HEI score were also considered, the relationship between total polyphenol intake and CAL was not detected. Total fiber intake was not associated with our inflammatory markers, though we found that the total HEI score negatively associated with CAL (*P* = 0.01). We did not observe a relationship between total polyphenol intake and our other inflammatory outcomes. However, several main effects were detected and are presented in detail in Table 4. Sex was predictive of CRP, LBP, and NEO, with males having a lower average CRP and LBP. BMI was a predictor of CRP and LBP, with a positive relationship observed with BMI. These relationships and their directionality with BMI and sex remained after accounting for fiber intake and total HEI.

**TABLE 4:** Summary of models examining the relationship between total polyphenol intake and inflammatory outcomes. Covariate relationships with strong evidence of a relationship with the outcome are listed. Rank-based estimation regression models were analyzed with age, sex, and BMI as predictors and with and without average total fiber intake and ASA24 total HEI score. Rank-based estimation regression models accounted for our main covariates of age, BMI, and sex (without fiber, total HEI). Additional models were analyzed which accounted for main covariates, total fiber intake, and HEI-2015 total score (with fiber, total HEI).

Includes fiber, total HEI	Marker	Total polyphenol β	Total polyphenol *P* value	Covariates relationships
No	CRP	−0.03	0.8	BMI β = 253, *P* < 0.001 Sex, male β = −1209, *P* < 0.001
Yes	CRP	−0.021	0.86	BMI β = 248, *P* < 0.001 Sex, male β = −1146, *P* < 0.001
No	LBP	0	0.81	BMI β = 0.472, *P* < 0.001 Sex, male β = −1.73, *P* < 0.001
Yes	LBP	0	0.9	BMI β = 0.479, *P* < 0.001 Sex, male β = −2.01, *P* < 0.001
No	CAL	−0.004	0.04^1^	
Yes	CAL	−0.003	0.12	HEI total score β = −0.434, *P* = 0.01
No	MPO	−0.026	0.07	
Yes	MPO	–0.025	0.09	
No	NEO	−2.21e–04	0.44	Sex, male β = −1.77, *P* < 0.01
Yes	NEO	−4.99e–05	0.87	Sex, male β = −1.431, *P* = 0.01

Abbreviations: BMI, body mass index; CAL, calprotectin; CRP, C-reactive protein; HEI, healthy eating index; LBP, lipopolysaccharide binding protein; MPO, myeloperoxidase; NEO, neopterin.

1 Strong evidence of a relationship between polyphenol intake and the given marker.

### Relations of polyphenol classes with inflammation

When we decomposed total polyphenol intake into underlying classes, we found that phenylpropanoic acids and prenol lipids were negatively associated with plasma LBP, of which BMI and sex were also important predictors (Figure 1). Associations held after adjustment for fiber intake and total HEI.

**FIGURE 1:**
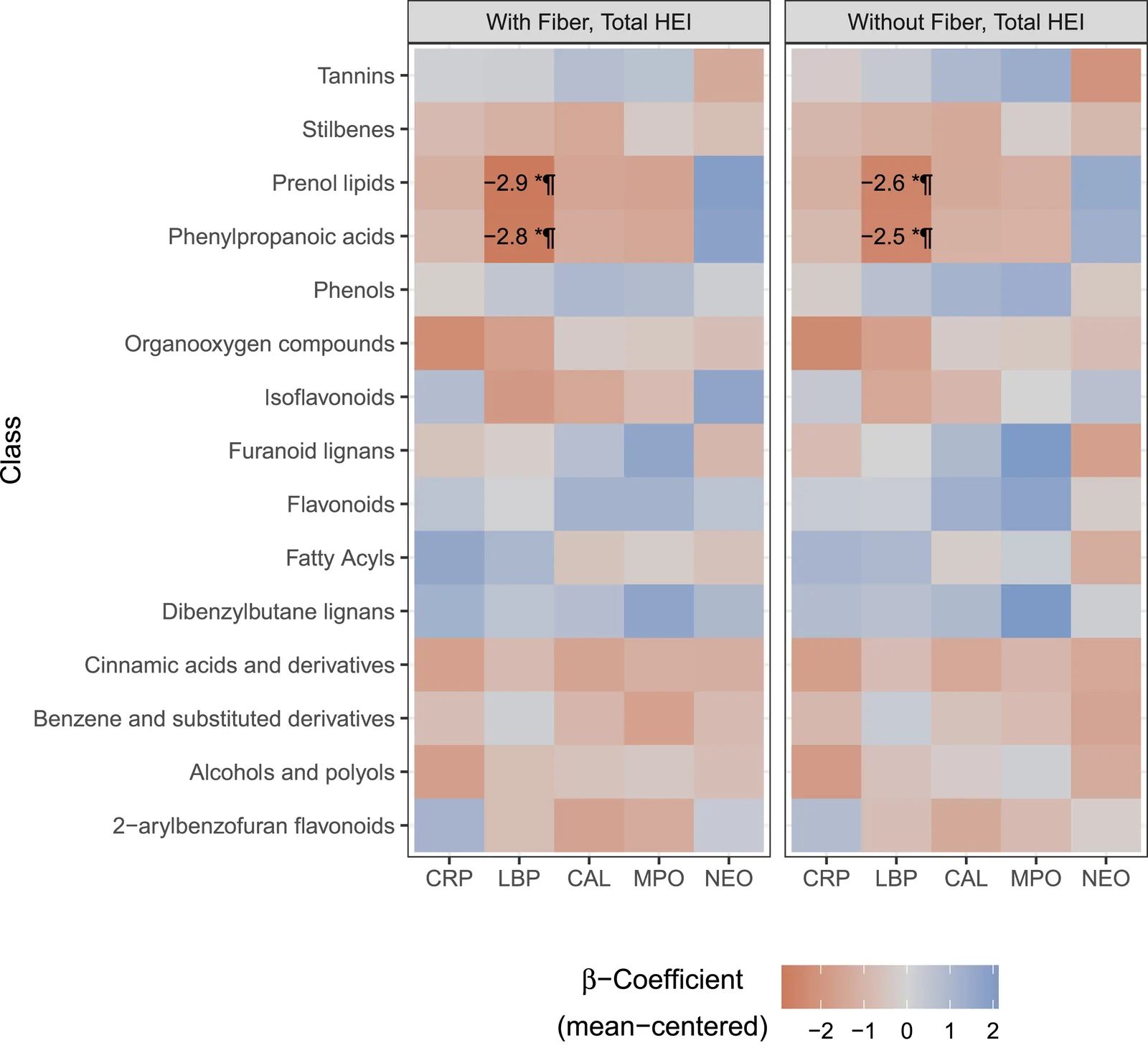
Relationships between polyphenol class and inflammation outcomes. Heatmap tile color represents the directionality of the relationship. Additional labels on heatmap tiles denotes polyphenol classes with strong evidence of a relationship with an inflammation outcome. Beta coefficients were derived from separate class-specific linear models, where polyphenol class, age, body mass index, and sex were predictors (without fiber and total HEI). Models were also performed additionally accounting for total fiber intake and HEI-2015 total score (with fiber and total HEI). *P* values were adjusted using the false discovery rate correction for multiple testing. For visualization purposes, all coefficients were normalized to the mean of the absolute coefficient values for each inflammation marker. Covariate effects for highlighted polyphenol classes are indicated as follows: ∗ for body mass index and ¶ for sex. CAL, calprotectin; CRP, C-reactive protein; HEI, healthy eating index; LBP, lipopolysaccharide binding protein; MPO, myeloperoxidase; NEO, neopterin.

The examination of intakes of phenylpropanoic acids and prenol lipids against LBP revealed similar intake patterns (Supplemental Figure 3). Intakes of both classes were primarily driven by consumption of olive products (Figure 2). Three phenylpropanoic acids were present in our estimates of polyphenol intake with only one compound quantified in FooDB (FDB008857: 3-(3,4-dihydroxyphenyl) propanoic acid) stemming solely from olive products. Seventy-six polyphenols were attributed to the prenol lipid class though only 7 were quantified in FooDB: oleuropein-aglycone, oleuropein, acetoside, demethyloleuropein, 3,4-DHPEA-EA, gossypol, and malvin. Gossypol was uniquely attributed to sunflower seeds, malvin was attributed to nectarines, and the remaining 5 polyphenols stemmed from olive products. Compared with olives, compounds from nectarines and sunflower seeds contributed relatively less to class intake. Average intakes of malvin and gossypol were, respectively, 0.16 mg from 55 consumers of sunflower seeds and 0.09 mg from 14 consumers of nectarines.

**FIGURE 2:**
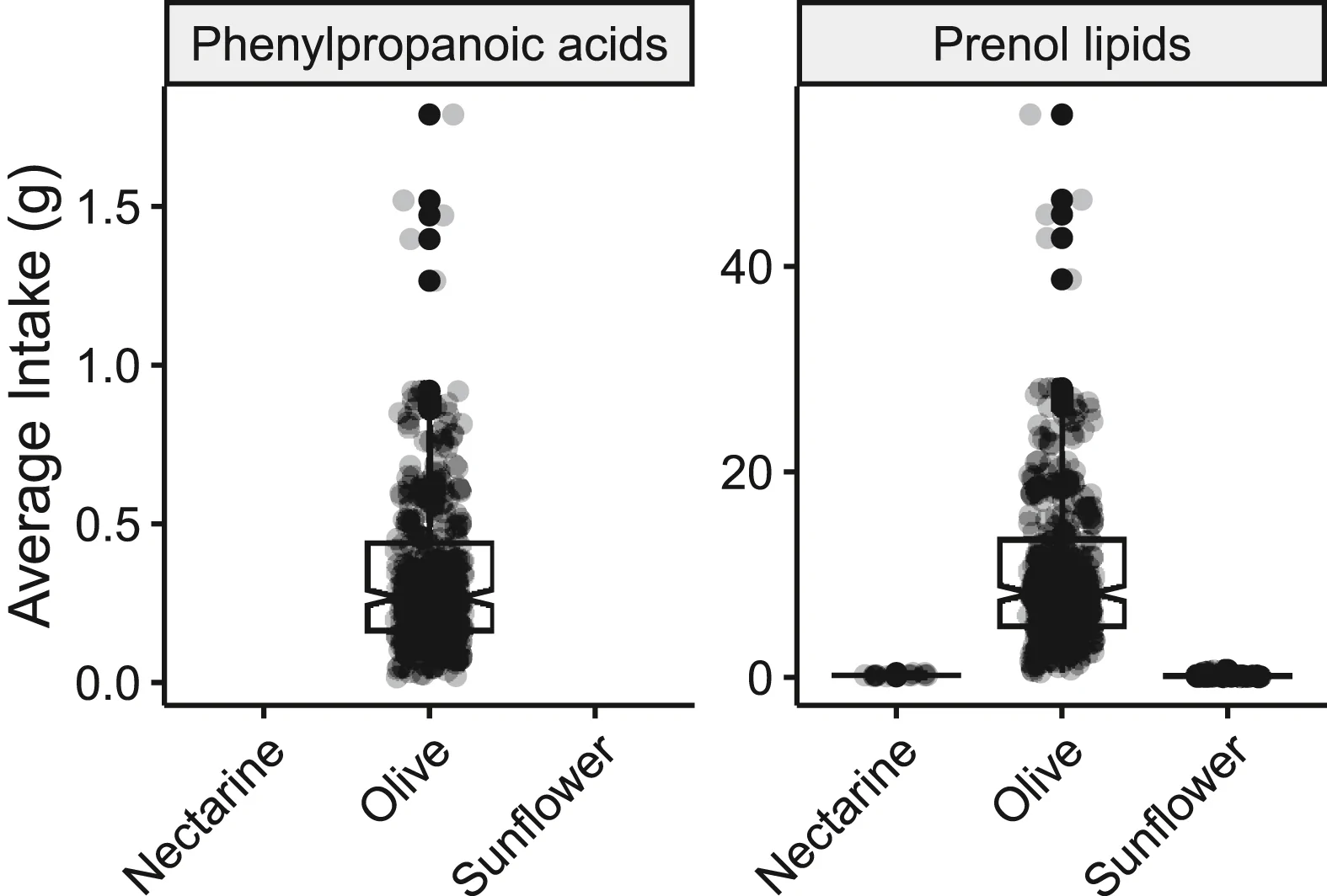
Food contributors to prenol lipids and phenylpropanoic acids intake. The x-axis represents foods within FooDB with nonzero quantification. The y-axis represents average daily intake of each food.

Two phenylpropanoic acids were unquantified in FooDB but predicted to occur in a variety of livestock and wild game animals. Additionally, 69 prenol lipids were unquantified in FooDB but predicted in a wide assortment of foods.

### Relations of polyphenol compounds with inflammation

To determine the relationships between polyphenol compounds and inflammation that may have been missed by directed hypothesis testing, we used hierarchical feature engineering to reduce the number of polyphenol features and then assembled and tested random forest regression models. Hierarchical feature engineering reduced the initial 1483 polyphenol features to 12 features for CRP and 54 features for CAL. Selected polyphenol features and study covariates (Supplemental Table 1) served as inputs for our random forest regression models.

BMI was the top feature in predicting the systemic inflammatory marker CRP, with a positive relationship observed (Figure 3A). The total HEI score was the top feature in predicting CAL, with a negative relationship observed (Figure 3B). Polyphenolic compounds and groups were present among top 5 features across all models. Performance metrics, including those for null model on inflammation markers, are provided in Supplemental Table 2.

**FIGURE 3:**
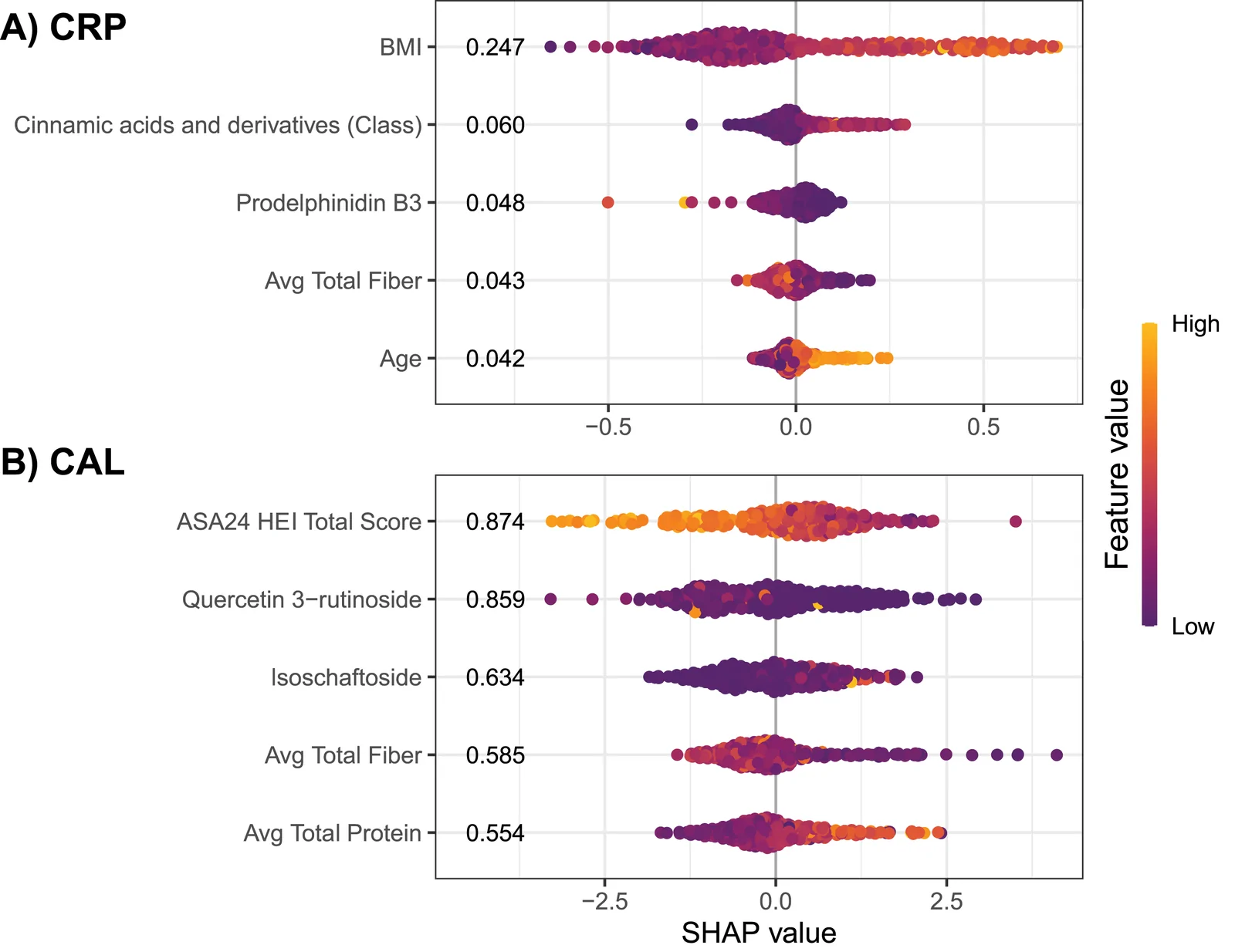
SHAP summary plots from random forest regression models on subclinical inflammation. The top 5 most important features for (A) CRP and (B) Fecal CAL are shown. Feature importance values are ordered from high to low on the left of each panel. Each point represents the individual contribution to the model output and is organized like a density plot. Color indicates the original value of the feature. CRP underwent a Box-Cox transformation, whereas calprotectin was on the original scale. BMI, body mass index; CAL, calprotectin; CRP, C-reactive protein; HEI, healthy eating index; SHAP, Shapley Additive exPlanations.

The beeswarm plot, when examined in conjunction with related correlation analyses, reveals a positive relationship between CRP and polyphenols classified as “Cinnamic acids and derivatives” and a negative relationship between CRP and total fiber intake (Figure 4A). In relation to CAL, a negative relationship was observed with total fiber intake and a positive relationship observed with protein intake (Figure 4B). Aside from “Cinnamic acids and derivatives,” observed relationships between polyphenols and inflammation markers within the top 5 features appear to be influenced by outlier polyphenol intakes. To examine the variation of the “Cinnamic acids and derivatives” estimate, we calculated the bootstrap confidence interval for the β-coefficient on CRP accounting for age, BMI, and sex. The 95% confidence interval for the β-coefficient ranges from 0.0050 to 0.0408.

**FIGURE 4:**
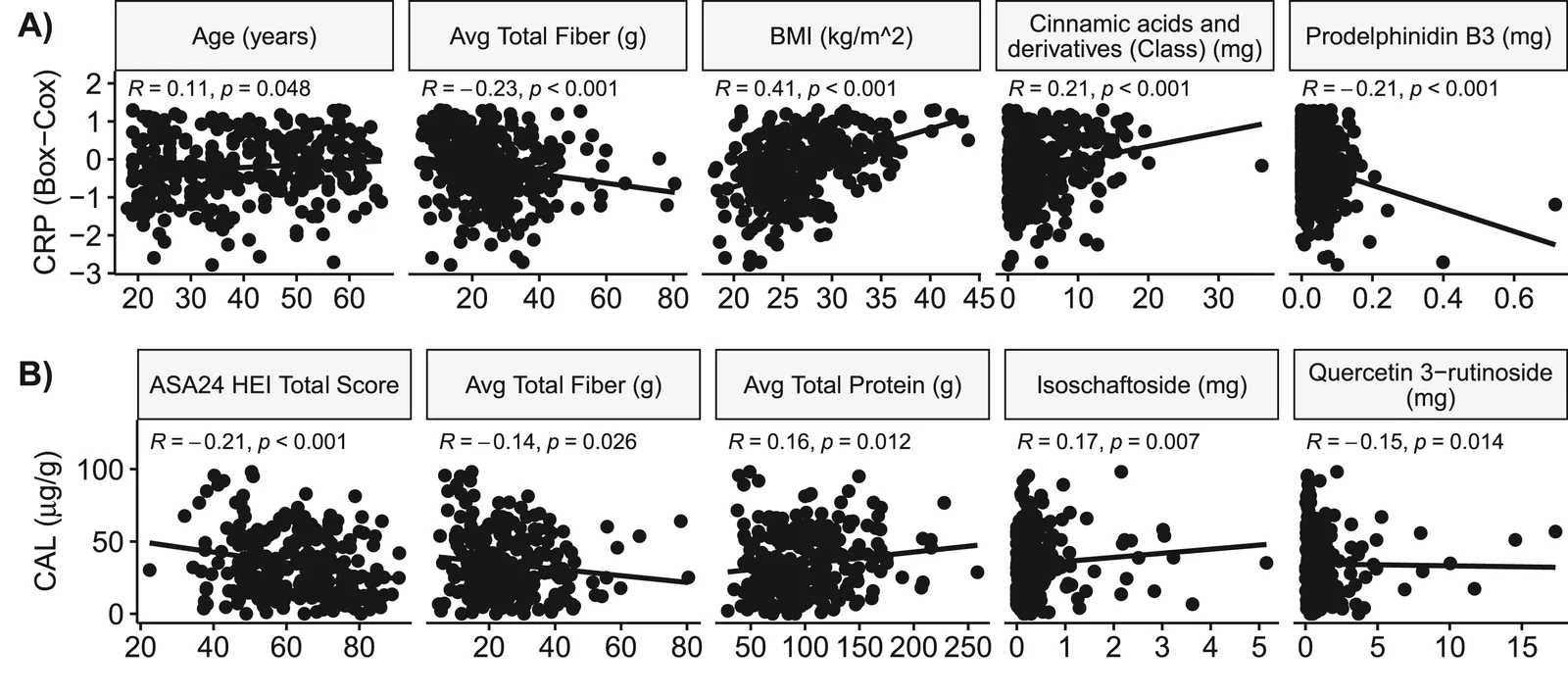
Spearman correlations between informative model features and subclinical (A) CRP and (B) Fecal CAL. Top 5 features from each random forest regression model are present, with feature importance determined by averaging absolute SHAP, values. Subclinical inflammation was defined as >10 mg/L for CRP and >100 μg/g for CAL. BMI, body mass index; CAL, calprotectin; CRP, C-reactive protein; HEI, Healthy Eating Index; SHAP, Shapley Additive exPlanations.

Fifty-three polyphenols classified as “Cinnamic acids and derivatives” were found in participant dietary data. Caffeic acid exhibited the highest average intake among the 20 quantified polyphenols in this class. Food contributors to “Cinnamic acids and derivatives” included coffee, apples, and potatoes. Common food sources of other polyphenol features are included in Supplemental Table 3.

## Discussion

We analyzed the relationship between dietary polyphenol intake (total, class, and individual polyphenols) and systemic and GI inflammatory markers, accounting for age, BMI, dietary quality, fiber intake, and sex. We found that total polyphenol intake negatively associated with the GI inflammation marker CAL (Table 4). Similarly, polyphenols classified as prenol lipids and phenylpropanoic acids negatively related to the GI permeability marker LBP (Figure 1), with intakes of these polyphenol classes primarily driven by consumption of olive products. We additionally utilized hierarchical feature engineering in combination with machine learning to examine the relationship between polyphenols and subclinical levels of inflammation. In this, we detected a positive relationship between “cinnamic acids and derivatives” and the systemic inflammation marker CRP (Figure 3). Our results suggest that polyphenol intake may additionally benefit indicators of GI health and that certain polyphenol classes may exert systemic proinflammatory effects.

Of top food contributors to total polyphenol intake in our healthy adult cohort, tea was the largest contributor followed by grapes, oranges, and several other fruits (Table 3). Many of our food contributors are widely considered polyphenol-rich foods which include spices and herbs, fruits, seeds, vegetables, and nonalcoholic beverages such as coffee and tea [42]. Wheat and beans are not commonly thought of as polyphenol-rich foods but have made it previously onto polyphenol contribution lists [6,43,44]. The contributions from wheat and beans that we detected are likely overestimated as food processing metadata was not complete to fully account for whole compared with unprocessed food forms. The average total polyphenol intake in our cohort was 914 mg per 1000 kcal/d, which is close to the estimate of 884.1 mg per 1000 kcal/d from an analysis of 2016–2017 National Health and Nutrition Examination Survey dietary data [44] but higher than the 801 mg/d predicted in a 2016-analysis of the Adventist Health Study-2 [43]. Flavonoids were the largest class contributor, with our mean flavonoid intake (495 mg per 1000 kcal/d) also higher than United States cohort studies (273–379 mg/d) [43,44]. Polyphenol intake in adults is dependent on personal and sociocultural food preferences with variation seen with geographical region [5]. The difference in these United States-based estimates may also partly stem from database differences as earlier estimates relied on older databases with less coverage.

Relationships between polyphenol intake and our inflammatory outcomes varied by the level at which polyphenol intake was summarized and analyzed. Fecal CAL was predicted by total polyphenols but not by polyphenol class or individual compounds, and LBP was predicted by two polyphenol classes but not by total intake or individual compounds. Total polyphenol intake, our broadest summary, was associated with lower intestinal inflammation indicated by CAL, which suggests that fecal CAL may be influenced by a collective polyphenol anti-inflammatory effect. One mechanism by which dietary polyphenols exert an anti-inflammatory effect is through inhibition of nuclear transcriptional factors in the inflammation-associated toll-like receptor 4 signaling pathway [13,14]. The relationship with CAL disappeared after accounting for overall diet quality, which was unsurprising as improving diet quality is a general recommendation to reduce inflammation [45]. At the class level, we observed lower LBP, a proxy for GI permeability, with greater intake of phenylpropanoic acids and prenol lipids. The relationship with LBP could be due in part to the bioavailability or bioefficacy of phenylpropanoic acids and prenol lipids or stem from other compounds within the food sources of these polyphenol classes. Intakes of these classes was primarily attributed to olive products such as ripe olives, pickled olives, and olive oil. Polyphenols within olives may potentially reduce LPS-induced inflammation and subsequent LBP production through inhibition of intracellular proinflammatory mediators [46]. Olive oil in particular is a hallmark of the Mediterranean diet, which in addition to its rich monounsaturated fatty acid profile, contains polyphenols that have garnered attention for their potential to improve cardiovascular health outcomes [47,48] and lower inflammation [49]. An overestimate of phenylpropanoic acids and prenol lipids is likely as olive products are typically incorporated into cooked dishes compared to consumed raw.

A surprising finding from our machine learning modeling was the positive association between polyphenols classified as “cinnamic acids and derivatives” and CRP. CRP is an acute phase protein mainly produced by hepatocytes in response to a range of inflammatory cytokines. Polyphenols have been negatively associated with CRP in epidemiological studies [50,51]. With polyphenol interventions, an analysis of the Healthy Lifestyle in Europe by Nutrition in Adolescence (HELENA) Study found that associations between polyphenols and inflammation are dependent on the type of polyphenol and inflammation biomarker [52]. The HELENA analysis highlights that individual polyphenols and polyphenol classes may have proinflammatory and anti-inflammatory relationships [52]. Improvements to CRP have not been found with whole food or polyphenol extract interventions [2]. In vitro research suggests that a proinflammatory effect may be partially because of the increased oxidative stress from an elevated polyphenol load [53,54]. Coffee was the largest contributor to “cinnamic acids and derivatives” intake and is generally associated with lower systemic inflammation in the United States [55]. It is possible that the positive relationship observed with CRP may stem from differences in dietary profiles between coffee consumers and noncoffee consumers [43], such as the addition of sugar and high-fat cream to coffee. Dietary cinnamic acids are commonly metabolized by gut microbes into their corresponding phenylpropanoic (dihydrocinnamic) acids [56], which warrants further exploration of the relationship between microbially produced and dietary phenylpropanoic acids on inflammation.

Our study has limitations. First, causal relationships cannot be made with our cross-sectional study design. Dietary interventions across geographic regions are needed to better assess the impact of increased food-based polyphenol intake on GI inflammation. Second, our machine learning models explain our dietary and inflammation data and are not generalizable. Third, this study works with the estimates of polyphenol intake that were not validated by measurement of polyphenol concentrations in biofluids. Diet recalls have sources of measurement error including recall bias [57]. Our ability to then map dietary recalls is constrained by what is present in the food composition database. For example, herbs and spices are polyphenol-rich ingredients [42] with limited coverage in FNDDS recipes. Increasing the coverage of herbs and spices within FNDDS recipes can help improve polyphenol reporting in the United States, especially among consumers of diverse cultural foods. Supplement and detailed food metadata (plant cultivars, growing conditions, and product brands) are also lacking, which contributes to the low resolution of polyphenol concentrations. Lastly, our intake estimation and analyses did not consider the contributions of phenolic acids (such as ferulic acid), which can display similar functionality to polyphenols or aspects of polyphenol bioavailability [7,58] and potential compound synergies from varied food matrices [59,60].

Our study has notable strengths. A key strength is the use of multiple 24-h recalls. Multiple dietary recalls were collected from participants within a 14-d period and went through an extensive cleaning process [29], allowing for improved assessment of a typical diet for each participant. In mapping our dietary data, we used a semiautomated method to disaggregate mixed dishes into underlying ingredients, which allowed for more specific food linkages to foundational foods within FooDB. Another strength is that FooDB allowed for polyphenol content to be estimated from multiple built-in food composition databases with polyphenols in addition to experimental data from publications detailing food polyphenols not well represented in food composition databases. These methods are in line with polyphenol intake estimation recommendations [18]. Additionally, there are few studies that have examined the relationship between GI inflammatory markers and polyphenol intake at the compound, class, and total summary levels. Our study helps address the current absence of information on how polyphenols may relate to GI inflammation and suggests that the relationship between inflammation and polyphenols should consider differential relationships between polyphenols.

In conclusion, we found tea, coffee, and fruits to be among the top contributors to polyphenol intake. We also found associations between dietary polyphenol intake and systemic and GI inflammatory markers. These associations varied across total polyphenols, polyphenol classes, and individual polyphenols. Future research with GI inflammation should consider disaggregated analyses of total polyphenol intake to better understand relationships with inflammation as polyphenol bioavailability and bioactivity is influenced, in part, by polyphenol structure. We also recognize that different individuals likely require different amounts of polyphenols for the same GI benefit because of the large variation in polyphenol metabolism by the host and the gut microbiome. Additional research is needed to identify the polyphenol degrading capacity of gut bacteria and their prevalence in the gut microbiome to better understand the bioavailability and bioefficacy of polyphenols and their derivatives.

## Acknowledgments

We acknowledge Dr. Ellen Bonnel for human studies management; Evelyn Holguin, Annie Kan, Barbara Gale, Danna Juarez Rios, Justin Waller, Ashley Tovar, Christine Bowlus, Yasmine Bouzid, Yanhua “Eva” Li, and Diane Han for human studies and physiology assessment in the USDA Nutritional Phenotyping Study; and Joanne Arsenault for dietary quality control.

## Author contributions

The authors’ contributions were as follows – JMA, DGL, CBS: designed research; ZA, SMGW: conducted research; SMGW, JAL, JJN: provided essential materials; SMGW, AO, DGL: analyzed data; SMGW, DGL: wrote the article; DGL: had primary responsibility for the final content; and all authors: read and approved the final manuscript.

## Conflict of interest

The authors declare that the research was conducted in the absence of any commercial or financial relationships that could be construed as a potential conflict of interest and report no conflicts of interest. CBS is a member of *The Journal of Nutrition’s* Editorial Board and played no role in the Journal’s evaluation of the manuscript.

## Funding

This research was supported in part by the Texas A&M Institute for Advancing Health Through Agriculture USDA-ARS Agreement 58-3091-1-018 USDA-ARS 2032-51530-026-000-D, USDA-ARS 2032-10700-002-000D and USDA-NIFA AI Institute for Next Generation Food Systems (AIFS), USDA-NIFA award number 2020-67021-32855. The project described was also supported by the National Center for Advancing Translational Sciences (NCATS), National Institutes of Health (NIH), through grant UL1 TR001860. USDA is an equal opportunity employer.

## Data availability

Requests for nonmetagenomic data from the USDA-ARS WHNRC Nutritional Phenotyping Study used in this analysis should be made via an e-mail to the senior WHNRC author on the publication of interest. Requests will be reviewed quarterly by a committee consisting of the study investigators. A list of FooDB polyphenols used in this study and scripts for our FooDB database mapping, polyphenol estimation, and analysis can be found on GitHub at https://github.com/SWi1/FooDB_polyphenol_analysis.
