# Supplementary material for: Fine-Scale Dietary Polyphenol Intake Is Associated with Systemic and Gastrointestinal Inflammation in Healthy Adults
Source: J Nutr. 2024 Aug 18;154(11):3286–97. doi: 10.1016/j.tjnut.2024.08.010 (PMC13639619; doi:10.1016/j.tjnut.2024.08.010)
Supplement: Supplementary file 1 [file mmc1.docx]

**Supplementary Materials for**

**Fine-scale Dietary Polyphenol Intake is Associated with Systemic and Gastrointestinal Inflammation in Healthy Adults**

Stephanie M.G. Wilson^1,2^, Andrew Oliver^1^, Jules A. Larke^1^, José J. Naveja^3,4^, Zeynep Alkan^1^, Joseph M. Awika^2^, Charles B. Stephensen^1,5^, Danielle G. Lemay^1,5*^

^1^ United States Department of Food and Agriculture, Agricultural Research Service Western Human Nutrition Research Center, Davis, California, USA

^2^ Texas A&M AgriLife Research, Institute for Advancing Health Through Agriculture, College Station, Texas, USA

^3^ University Medical Center, Johannes Gutenberg University Mainz, Mainz, Germany

^4^ Institute of Molecular Biology gGmbH, Mainz, Germany

^5^ Department of Nutrition, University of California, Davis, Davis, California, USA

Contents:

Supplementary Figures 1-3

Supplementary Tables 1-3

**Supplementary Figures**

Supplementary Figure 1. **A flowchart of the study design.** Of the 393 adults who enrolled into the study (completed the first visit), 350 participants completed the full study and had dietary recalls which passed quality control.

Supplementary Figure 2. **Database handling and mapping schema for the quantification of polyphenol content using ASA24 recalls and FooDB.** Blue text indicates important action taken. Keys denote variable names used to map between databases. Food and compound numbers represent the unique number of each within a given data frame.

Supplementary Figure 3. **Regression plots of polyphenol classes with lipopolysaccharide binding protein**. Blue line indicates Spearman correlation coefficient. For phenylpropanoic acids, intake was solely from olive products. For prenol lipids, intake was from largely from olive products with minor contributions from sunflower seeds and nectarines. Points highlighted in red indicate subjects who were consumers of sunflower seeds and nectarines. Abbreviations: LBP, lipopolysaccharide binding protein.

**Supplementary Tables**

Supplementary Table 1. **Summary of Machine Learning Variables for Subclinical Inflammation**. Inputs listed under ‘All’ were inputs into each random forest regression model. Model-specific polyphenol additions are noted below with polyphenol inputs determined by hierarchical feature engineering using taxaHFE. Abbreviations: BMI, body mass index; CAL, calprotectin; CRP, C-Reactive Protein; HEI, healthy eating index; LBP, lipopolysaccharide binding protein; MPO, myeloperoxidase; NEO, neopterin.

| Outcome | Inputs |
| --- | --- |
| All | Age (years)  Sex (M/F)  BMI (kg/m^2^)  Total Fiber Intake (g)  Total HEI Score (0-100)  Total Protein Intake (g)  Education Level (Primary School, Junior high or middle school, high school/GED/equivalent, Some College/Associate degree, Bachelors, Graduate/non-doctoral, Doctorate) |
| + CRP | Cinnamic acids and derivatives, Gentisic acid (FDB000844), 1,2-Benzenediol (FDB001512), 3,5-Di-O-caffeoylquinic acid (FDB002628), Hyperin (FDB002711), Quercetin (FDB011904), Valoneic acid dilactone (FDB000230), 5-Pentacosenylresorcinol (FDB000355), Pelargonidin 3-glucoside (FDB002748), Prodelphinidin B3 (FDB016774), Luteolin 7-glucuronide (FDB017828), Theogallin (FDB018832) |
| + CAL | Organic acids and derivatives, Lipids and lipid-like molecules, Organic oxygen compounds, Cyclobutane lignans, Indanes, Isorhamnetin (FDB000604), Kaempferol (FDB000633), Kaempferol 3-glucuronide (FDB000634), 6''-O-Acetylgenistin (FDB000669), 6''-Malonylgenistin (FDB000670), Gentisic acid (FDB000844), 1,2-Benzenediol (FDB001512), Quercetin 3-rutinoside (FDB002536), Cyanidin (FDB002602), 3,5-Di-O-caffeoylquinic acid (FDB002628), Hyperin (FDB002711), Malvidin 3-glucoside (FDB002714), Hydroxytyrosol (FDB008754), 4-Methyl-1,2-benzenediol (FDB008861), 8-Acetoxypinoresinol (FDB011303), Coumestrol (FDB011663), Genistein (FDB011828), Naringin (FDB011866), Quercitrin (FDB011884), Quercetin (FDB011904), Dopamine (FDB012163), Procyanidin B3 (FDB012205), Luteolin (FDB013255), Oleacein (FDB016341), Apigenin 7-glucoside (FDB016362), Pelargonidin 3-(6''-succinyl-glucoside) (FDB000014), Malvidin 3-(6''-caffeoyl-glucoside) (FDB000055), Procyanidin trimer EEC (FDB000108), 5-5'-Dehydrodiferulic acid (FDB000263), 3,5-Diferuloylquinic acid (FDB000277), delta-Viniferin (FDB000319), Hydroxytyrosol acetate (FDB000344), 3- |
| Outcome | Inputs |
| + CAL (cont.) | Methylcatechol (FDB000357), 5,3',4'-Trihydroxy-3-methoxy-6,7-methylenedioxyflavone 4'-glucuronide (FDB001550), Isoschaftoside (FDB002645), Malvidin (FDB002713), 1,2,3-Benzenetriol (FDB008735), Ethyl gallate (FDB012004), Procyanidin B4 (FDB012206), Genistin (FDB012221), Isoorientin (FDB012395), Isolariciresinol (FDB012663), Luteolin 7-glucoside (FDB014280), Patuletin 3-(2''-apiosylgentiobioside) (FDB016624), Prunin (FDB016683), 4-Ethyl-1,2-benzenediol (FDB019886), Isorhamnetin 3-O-b-D-glucopyranoside (FDB021629), 3,4',5,6,7,8-Hexahydroxyflavone (FDB021746), Dihydromyricetin 3-rhamnoside (FDB021841) |

Supplementary Table 2. **Descriptive statistics and** **performance metrics of random forest regression models on subclinical inflammation outcomes. ***CRP (ng/mL) underwent a Box-Cox transformation. Abbreviations: CAL, calprotectin; CRP, C-Reactive Protein; MAE, mean absolute error.

|  | Descriptive Statistics | | | | |  | Performance Metrics | | |
| --- | --- | --- | --- | --- | --- | --- | --- | --- | --- |
| Marker | Count | Mean | Std. Err. | Min | Max |  | MAE, RF | R2, RF | MAE, null |
| CRP* | 260 | 34.4 | 1.4 | 0 | 98.2 |  | 0.609 | 0.136 | 0.674 |
| CAL (μg/g) | 310 | -0.2 | 0 | -2.8 | 1.3 |  | 15.804 | 0.08 | 18.005 |

Supplemental Table 3. **Polyphenol features from machine learning regression models and their common food sources.** Associations with inflammation markers and their directionality is provided, with + indicating higher inflammation and – indicating lower inflammation. Feature directionality determined by average Shapley value. Abbreviations: CAL, calprotectin; CRP, C-reactive protein.

| **Taxon or Compound** | **Common Food Sources** | **Associated Markers (Direction)** |
| --- | --- | --- |
| Cinnamic acids and derivatives (Class) | Coffee, Apple, Potato | CRP (+) |
| Isoschaftoside | Soybean | CAL (+) |
| Prodelphinidin B3 | Tea, Beer, Grape wine | CRP (+) |
| Quercetin 3-rutinoside | Parsley, Apple, Spinach | CAL (-) |
